# Proliferative verrucous leukoplakia: a general dental practitioner-focused clinical review

**DOI:** 10.1038/s41415-024-7066-8

**Published:** 2024-02-23

**Authors:** Konrad Staines, Helen Rogers

**Affiliations:** 4141530895001 Consultant Oral Medicine and Honorary Professor, Department of Oral Medicine, Bristol Dental Hospital, Lower Maudlin Street, Bristol, BS1 2LY, UK; 4141530895002 Consultant Oral Medicine and Honorary Senior Clinical Lecturer, Department of Oral Medicine, Bristol Dental Hospital, Lower Maudlin Street, Bristol, BS1 2LY, UK

## Abstract

Proliferative verrucous leukoplakia (PVL) is a distinct type of oral leukoplakia which has the potential to enlarge or develop into new areas of leukoplakia coupled with areas of a warty surface texture. PVL is usually diagnosed from the fifth decade onwards and is more common in female patients. The most frequent sites involved tend to be gingivae, followed by buccal mucosa and lateral border of tongue. It is one of the oral potentially malignant conditions with a high risk of malignant transformation. It is important for general dental practitioners (GDPs) to identify such lesions to facilitate referral for further investigation and diagnosis. Management is challenging with long-term monitoring and surgical excision when appropriate; however, PVL tends to recur following surgical excision. This article provides an up-to-date review tailored for GDPs on the present knowledge of PVL and illustrates the management challenges with clinical cases.

## Key points

- Proliferative verrucous leukoplakia (PVL) is an important diagnosis due to the risk of malignant transformation.
- General dental practitioners are well placed to identify PVL in clinical practice and initiate early referral to specialist care.
- Long-term follow-up is required for patients with PVL.

## Introduction

Oral leukoplakia (OL) is a term which most general dental practitioners (GDPs) encounter in their clinical practice which describes white oral mucosal lesions of questionable risk.^1^ OL is defined by the 2005 World Health Organisation (WHO) as ‘a white plaque of questionable risk having excluded (other) known diseases or disorders that carry no increased risk for cancer'.^2^

In 1985, Hansen described an entity termed as a proliferative verrucous leukoplakia (PVL).^3^PVL is a distinct type of OL that GDPs are likely to encounter in their clinical practice and hence should be familiar with. The term ‘proliferative' describes the potential to enlarge or develop new areas of leukoplakia coupled with a tendency to recur following surgical excision.^4^ The term ‘verrucous' refers to the rough, warty surface texture that is often encountered in some areas of PVL.^5^

Over the last 20 years there has been debate in the academic literature as to whether the term PVL should be modified to multifocal,^6^ while others suggested deleting verrucous, only retaining ‘proliferative leukoplakia'.^7^ The WHO Collaborating Centre decided to retain PVL as its preferred terminology.^2^ PVL presents or at least tends to be diagnosed from the fifth decade onwards and is more common in female patients.^8^^,^^9^ The most frequent sites involved tend to be gingivae, followed by buccal mucosa and lateral border of tongue.^10^

The importance of recognition and appropriate management relating to this condition is particularly relevant as PVL is one of the oral potentially malignant disorders (OPMD).^11^ It is important to emphasise that not all patients with one of the OPMD will develop an oral malignancy; however, the diagnosis implies an increased risk compared to patients with clinically normal oral mucosal tissues.^2^ This has implications in terms of close monitoring over the longer term^4^and underscores the importance for GDPs to identify such lesions to facilitate referral for further investigation, diagnosis and management. This article provides an up-to-date review tailored for GDPs on the present knowledge of PVL.

## Aetiology of PVL

There is no single aetiological factor associated with PVL development. Indeed, in comparison to OL, the evidence linking consumption of tobacco and alcohol seems to be weaker.^12^^,^^13^ Attempts to associate other potential aetiological factors, such as human papilloma viruses, have not resulted in any firm evidence to confirm or exclude a potential role.^14^

## PVL and malignancy

PVL can transform into either an oral squamous cell carcinoma (OSCC) or an oral verrucous carcinoma (OVC).^15^ The identification of one or several areas of PVL not only represents a risk for the clinically altered tissue, but also indicates that there is an additional increased risk of developing a malignancy in clinically normal oral mucosal tissue.^12^ The reported malignant transformation (MT) rate of PVL varies between case series, with one significant systematic review and meta-analysis reporting that among 474 individuals with PVL, 211 (44.5%) underwent MT.^16^ Another systematic review reinforces the high rate of MT with an estimated yearly MT rate of 9.3% and an overall MT of 49.5%.^8^This compares to an overall MT rate of 7.9% for all OPMD.^8^ This places PVL at a significantly higher MT risk.^17^^,^^18^

In terms of site, the gingivae are the most common site where OSCC develops in pre-existing PVL, followed by the buccal mucosa and thereafter, lateral border of tongue.^10^ This contrasts with OL, where the lateral border of tongue is the highest risk site.^19^

Patients with PVL who develop an OSCC malignancy are at higher risk of developing a further primary OSCC, which can also be explained by the field cancerisation theory.^10^ On a more positive note, PVL-derived OSCC have better outcomes than conventional OSCC, with most patients surviving at the five-year mark.^18^ This can be explained by the smaller size of tumour and less likely lymph node metastases.^11^^,^^18^

Over the last decade, there have been efforts to identify one or more tissue-based biomarkers which would help estimate transformation risk.^19^ Unfortunately, to date, no single tissue-based biomarker can be relied upon consistently to help determine the risk of malignant transformation within PVL in routine specialist clinical practice.^20^

## Diagnosis of PVL

The academic debate over the criteria for PVL diagnosis spills over into the clinical domain, with differing opinions among clinicians when differentiating between OL and PVL.^5^^,^^7^ The diagnosis of PVL is primarily clinical with conventional visual inspection complemented by histopathological assessment.^9^ The histological picture is generally non-specific with a range of findings, such as hyperkeratosis, verrucous hyperplasia and dysplasia being the most common findings.^21^ Differentiation of PVL from OL is based on correlation of clinical findings, such as multifocal involvement of oral tissues, predilection for buccal mucosal, lingual and gingival sites, and verrucous or nodular surface texture coupled with a progressive onward expansion of the lesion.^3^^,^^22^ The criteria outlined in Table 1 can serve as a guide to diagnosis, with factors such as a leukoplakia with a verrucous surface involving more than two different oral sites, lesions which have spread, and sites typically involving the gingiva, alveolar processes and palate, being factors to consider in differentiation of a PVL from OL.^5^

**Table 1:** Criteria used for a diagnosis of PVL. In order to make the diagnosis of PVL, it was suggested that one of the two following combinations of the criteria were met: 1) three major criteria (E among them) or 2) two major criteria (E among them) + two minor criteria^5^

	Major criteria	Minor criteria
A	Leukoplakia lesion with more than two different oral sites, which is most frequently found in the gingiva, alveolar processes and palate	An OL lesion that occupies at least 3 cm when adding all the affected areas
B	The existence of a verrucous area	That the patient be female
C	That the lesions have spread or engrossed during development of the disease	That the patient (male or female) be a non-smoker
D	That there has been a recurrence in a previously treated area	A disease evolution higher than five years
E	Histopathologically, there can be from simple epithelial hyperkeratosis to verrucous hyperplasia, verrucous carcinoma or oral squamous cell carcinoma, whether *in situ* or infiltrating	

##  Visual diagnostic aids and PVL

Current evidence suggests poor specificity of diagnostic aids based on chemiluminescence or autofluorescence, which limits the clinical utility of these adjuncts for PVL diagnosis and monitoring.^23^ An alternative visual diagnostic aid is toluidine blue staining with evidence, albeit on OL not PVL, of a high sensitivity for the detection of carcinoma or carcinoma* in situ*.^24^

## Management of PVL in primary care

Patients with suspected PVL seen in general dental practice should be referred to the local oral medicine department (if available within the area) or alternatively, to the local oral and maxillofacial department on a routine or urgent basis, depending on clinical findings. The presence of signs such as marked surface irregularity/nodularity speckling, induration, or ulceration should result in escalation to a two-week wait referral. This referral standard is being revised to the Faster Diagnosis Framework and the Faster Diagnostic Standard from 1 October 2023.^25^GDPs are advised to include all relevant factors in the referral and, additionally, send high-quality clinical photographs which will help the triaging clinician.^1^ Invariably, following clinical assessment, tissue sampling via biopsy with histopathological assessment of one or more sites is routinely indicated and we would advise that such investigations are carried out in secondary care.^22^

### Learning point

If concern exists about a potential malignancy within the PVL, then a referral under an NHS two-week wait cancer pathway referral would be the appropriate referral pathway in the UK.

## Management of PVL in secondary care

The challenges around diagnosis and the progressive and generally extensive nature of PVL make it difficult to treat. There is a lack of evidence-based information regarding the optimal management of PVL patients and studies are often limited to small case series.^26^

### Medical approaches

There have been different forms of medical management considered for OL; however, the progressive and high malignant transformation rate make PVL difficult to manage.

Immunomodulatory agents have been explored for the management of PVL, although the numbers of patients in these studies have been small and re-occurrence occurred, requiring further surgery.^27^

### Surgical approaches

Surgical excision is often considered as the preferred management of patients presenting with PVL however this approach is complicated by a high rate of re-occurrence requiring further procedures. The different surgical techniques may include laser ablation, scalpel excision, electrocautery, cryoablation or photodynamic therapy.^26^ A study of OL managed by laser surgery showed a high rate of reoccurrence within the PVL subgroup requiring further surgical procedures.^28^

### Prevention

Different chemo preventative therapies have been studied for prevention of PVL, including retinoids, vitamin A, antioxidants and cyclooxygenase inhibitors but none have been shown to be clearly effective.^26^

Overall, early diagnosis, surveillance and regular consideration of biopsies would appear to be the most important strategy in the management of PVL patients.^11^

## PVL cases

The following cases demonstrate some of the most frequent demographic characteristics of PVL patients, that is, female with no history of alcohol or tobacco consumption. They demonstrate the progressive nature of PVL with the need for close surveillance to monitor for malignant transformation as demonstrated in Case 1. Female patients have been shown to be approximately three times more likely to develop OSCC than in male PVL patients.^11^ A patient with PVL may require an average of nine biopsies during their lifetime.^26^

### Case 1

A patient in their sixties was referred to the oral medicine department in Bristol Dental Hospital for a second opinion regarding a four-year history of OL and oral lichen planus. The concern was centred around white lesions progressively expanding with a marked irregular surface around the upper labial gingivae.

**Fig. 1:**
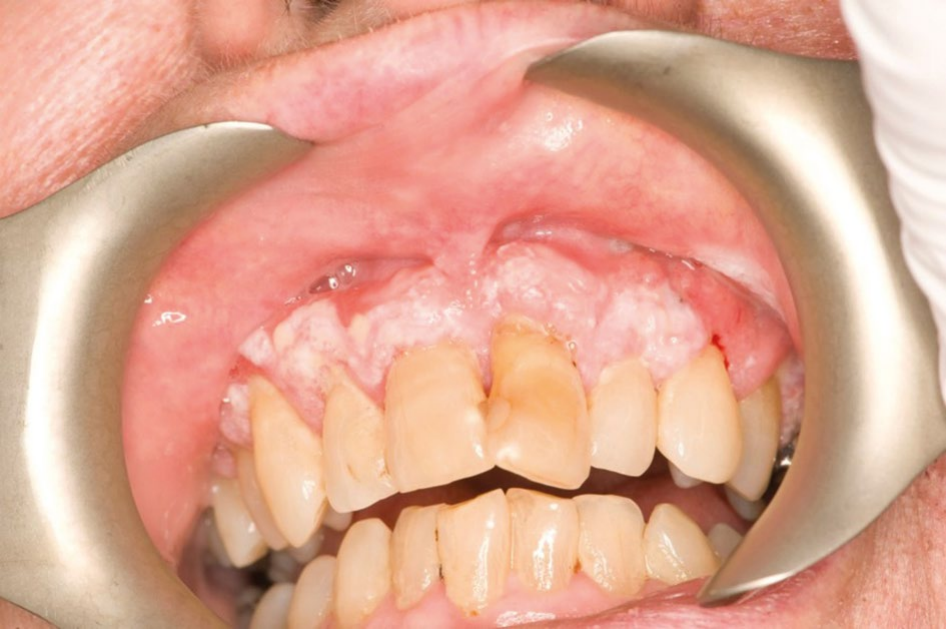
Clinical image of the upper labial gingivae. Reproduced with permission from Staines *et al.*, ‘Oral leukoplakia and proliferative verrucous leukoplakia: a review for dental practitioners', *British Dental Journal*, 2017, Springer Nature.^1^ The Licensed Material is not part of the governing OA license but has been reproduced with permission

Clinical findings on examination were consistent with a prominent verrucous white lesion involving the majority of the labial gingivae extending from upper canine to canine (Fig. 1).

The initial clinical impression was that of a PVL with a differential of OSCC or OVC. Three incisional biopsies were performed, with one of the samples resulting in histopathology consistent with verrucous squamous cell carcinoma, while the other samples yielded features consistent with verrucous hyperkeratosis and dysplasia. The patient was referred for further surgical management to oral and maxillofacial surgery.

### Case 2

**Fig. 2:**
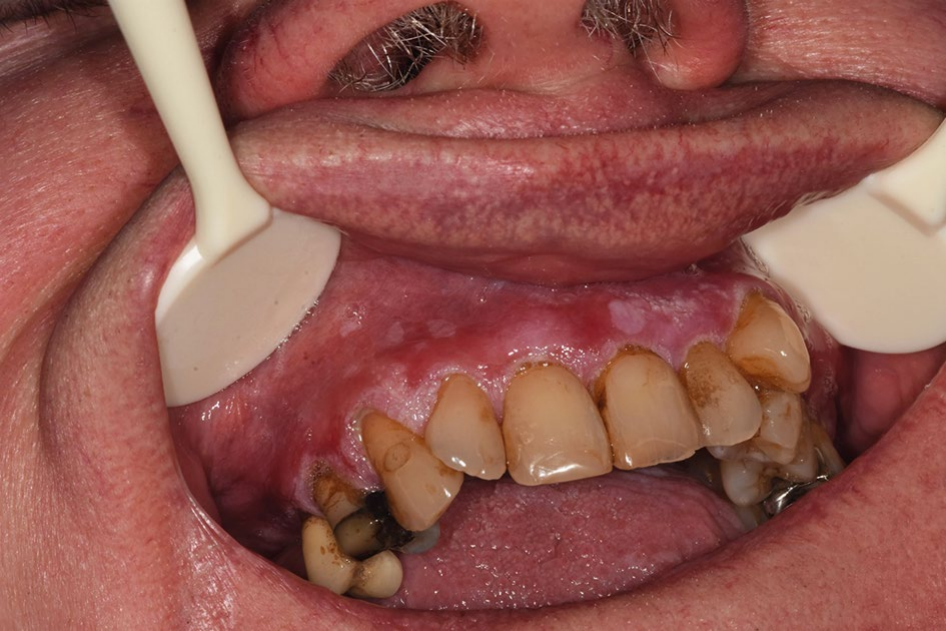
Clinical image of the upper anterior labial gingivae

**Fig. 3:**
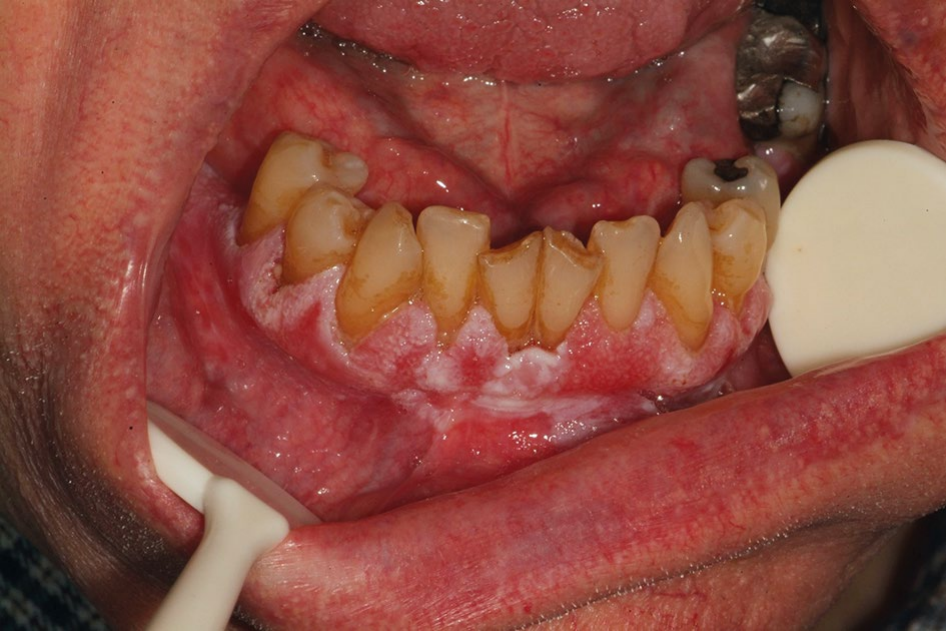
Clinical image of the lower right labial gingivae

A patient in their seventies was referred to oral medicine with a history of OLP and more recently, marked verrucous keratosis involving the gingival tissues (Fig. 2, Fig. 3). They were known to have atrial fibrillation, type 2 diabetes mellitus and were a non-smoker and consumed minimal alcohol. The appearance was consistent with PVL. A year later, the patient developed a localised exophytic lesion on the interdental papillae of the 42 and 43 region. An incisional biopsy report was that of a squamoproliferative lesion with verrucous architecture. Following MDT discussion, surgical excision of the lesion, including removal of their lower teeth, was undertaken. Excision of the lesion revealed an early invasive OSCC of the lower mandibular alveolar ridge.

The patient was subsequently followed-up on a regular basis. Five years after their initial OSCC, they developed a second OSCC involving the anterior hard palate, which involved excision with a maxillary alveolectomy. The patient continues to be followed-up at regular intervals.

### Case 3

**Fig. 4:**
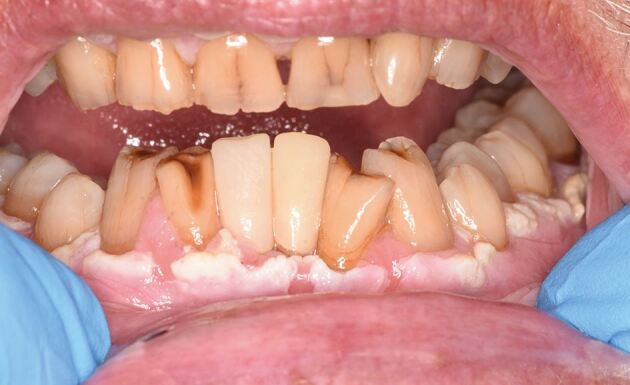
Clinical image of the lower anterior buccal gingivae

**Fig. 5:**
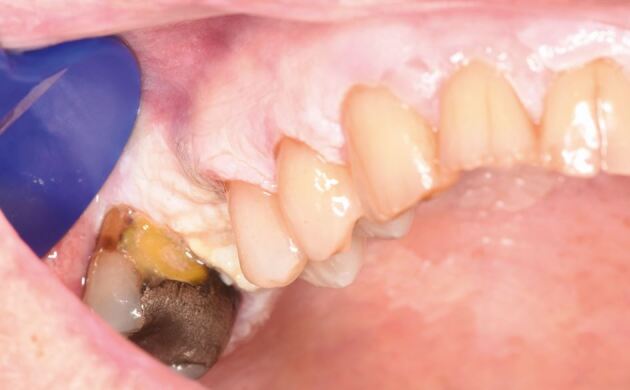
Clinical image of the upper right buccal gingivae

**Fig. 6:**
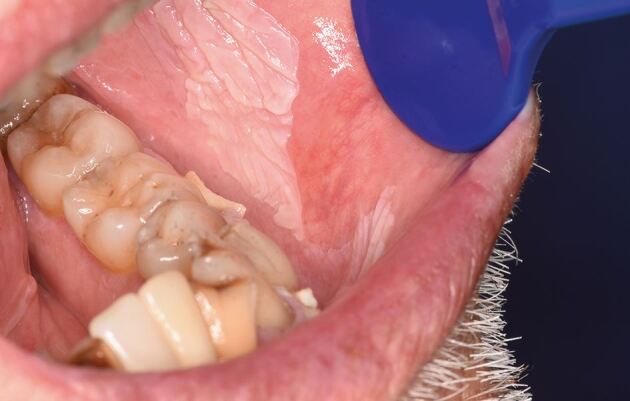
Clinical image of the lower left buccal mucosa

**Fig. 7:**
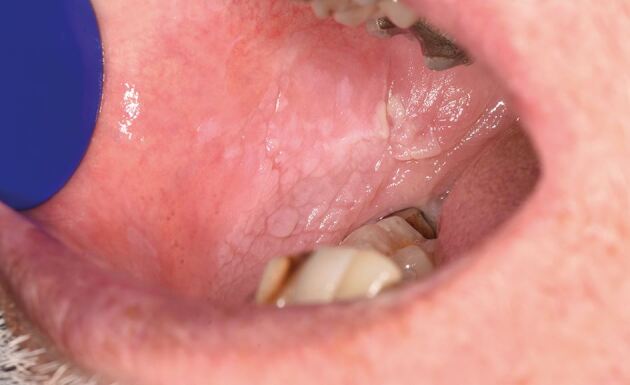
Clinical image of the lower right buccal mucosa

A patient in their seventies was referred to the oral medicine clinic for areas of gingival keratosis and an initial biopsy showed a verroucous morphology. An early diagnosis of PVL was made. They had a complex medical history of known chronic lymphocytic leukaemia and non-Hodgkin's lymphoma and was managed by the haematology team. Over a period of 12 years, they have been followed-up regularly by the oral medicine department with repeat biopsies when necessary. Over this review period, areas of keratosis have become more extensive, from affecting the buccal gingivae of the molar/premolar teeth in the mandibular arch as well as the buccal mucosa, to now involving the buccal gingivae in the maxillary arch and areas of lingual and palatal involvement (Fig. 4, Fig. 5, Fig. 6, Fig. 7).

## Conclusion

PVL is a significant clinical diagnosis which can be challenging to establish in dental practice. GDPs should consider the implications of potential MT.^29^ Prompt diagnosis and referral to secondary care services is indicated with all suspected PVL cases. Long-term monitoring is required for these patients^13^ and in some cases, active surgical management may be indicated; however, due to the extent of tissue involvement and field change implications, discussed above, surgical options are limited, while medical management currently is not supported by evidence in terms of efficacy.^11^
